# Effect of opioid prescribing guidelines in primary care

**DOI:** 10.1097/MD.0000000000004760

**Published:** 2016-09-02

**Authors:** Jonathan H. Chen, Jason Hom, Ilana Richman, Steven M. Asch, Tanya Podchiyska, Nawal Atwan Johansen

**Affiliations:** a Division of General Medical Disciplines, Department of Medicine, Stanford University, Stanford; b Center for Innovation to Implementation (Ci2i), Veteran Affairs Palo Alto Health Care System, Palo Alto; c Center for Primary Care and Outcomes Research (PCOR); d Department of Health Research and Policy—Epidemiology, Stanford University, Stanford, CA.

**Keywords:** drug screening, guidelines, noncancer pain, opioid, primary care

## Abstract

Long-term opioid use for noncancer pain is increasingly prevalent yet controversial given the risks of addiction, diversion, and overdose. Prior literature has identified the problem and proposed management guidelines, but limited evidence exists on the actual effectiveness of implementing such guidelines in a primary care setting.

A multidisciplinary working group of institutional experts assembled comprehensive guidelines for chronic opioid prescribing, including monitoring and referral recommendations. The guidelines were disseminated in September 2013 to our medical center's primary care clinics via in person and electronic education.

We extracted electronic medical records for patients with noncancer pain receiving opioid prescriptions (Rxs) in seasonally matched preintervention (11/1/2012–6/1/2013) and postintervention (11/1/2013–6/1/2014) periods. For patients receiving chronic (3 or more) opioid Rxs, we assessed the rates of drug screening, specialty referrals, clinic visits, emergency room visits, and quantity of opioids prescribed.

After disseminating guidelines, the percentage of noncancer clinic patients receiving any opioid Rxs dropped from 3.9% to 3.4% (*P* = 0.02). The percentage of noncancer patients receiving chronic opioid Rxs decreased from 2.0% to 1.6% (*P* = 0.03). The rate of urine drug screening increased from 9.2% to 17.3% (*P* = 0.005) amongst noncancer chronic opioid patients. No significant differences were detected for other metrics or demographics assessed.

An educational intervention for primary care opioid prescribing is feasible and was temporally associated with a modest reduction in overall opioid Rx rates. Provider use of routine drug screening increased, but overall rates of screening and specialty referral remained low despite the intervention. Despite national pressures to introduce opioid prescribing guidelines for chronic pain, doing so alone does not necessarily yield substantial changes in clinical practice.

## Abstract 2

Supplemental Digital Content is available in the text

## 1 Introduction

Drug overdose is now the leading national cause of accidental death, primarily driven by a public health epidemic^[1]^ of prescription (Rx) opioids that result in more unintentional deaths than cocaine and heroin combined.^[2]^ This may not be surprising given the 10-fold increase in opioid Rxs over the past 20 years,^[3]^ despite an overall lack of evidence to support their use in chronic noncancer pain.^[4]^ We now increasingly recognize the unintended consequences of this surge in Rxs, including diversion and misuse.^[5]^ Multiple groups and medical societies have put forth guidelines and recommendations for chronic opioid prescribing practices to address such issues.^[6–9]^ Recommendations include a preference for nonopioid treatment, screening for depression and substance abuse, defining functional treatment goals and a discontinuation plan, performing ongoing risk versus benefit assessments, avoiding concurrent benzodiazepine Rxs, use of Rx drug monitoring programs, prudent use of urine drug screening, offering medication-assisted therapy^[10]^ to those who develop an opioid use disorder, and seeking additional specialty help in those prescribed high daily morphine equivalents. Nonetheless, there is limited evidence supporting the effectiveness of many such approaches.^[11]^ Findings from workers’ compensation^[12]^ and health information technology–focused studies^[13]^ show promising associations between guideline implementations and lower overall opioids prescribed, with increased process compliance such as drug testing and functional assessments. However, these prior studies do not inform general practitioners on what to expect from introducing such guidelines and education into their practice. This can be particularly vexing given that educational interventions are typically ineffective at changing behavior.^[14]^

Recognizing that the vast majority of opioid Rxs still derive from general practitioners in primary care settings,^[15,16]^ we evaluated the introduction of an opioid prescribing guideline into multiple primary care clinics.

## 2 Methods

### 2.1 Guideline and protocol development

A multidisciplinary panel of Stanford clinicians formed an opioid working group to draft, revise, and consolidate comprehensive guidelines in September 2013 for treatment of patients with chronic noncancer pain. Input was integrated from providers in family medicine, internal medicine, pain medicine, psychiatry, and addiction medicine during both formal meetings and electronic correspondence. The relevant primary literature was reviewed, as were existing national guidelines from general medicine and subspecialist groups. Selected guideline recommendations are highlighted below (complete document in Supplementary Materials including reference to an Opioid Risk Tool^[17]^).

- Patients on opioids for chronic noncancer pain should be seen at least once every 3 months.
- Patients should be referred to pain clinic if demonstrating large, complex, or aberrant opioid consumption behavior.
- Patients should be referred to see a physical therapist if they are likely to benefit based on their particular pain diagnosis (Dx).
- Patients with chronic pain and comorbid psychiatric illness should be referred to a psychiatrist.
- Patients should undergo routine urine toxicology screening in clinic to monitor for drugs of abuse as well as possible diversion.
- Patients should receive their opioids from a single provider (their primary care provider) rather than multiple providers or emergency departments.
- When clinically appropriate, gradual attempts to reduce and eventually discontinue opioid use should be encouraged.

### 2.2 Intervention

These guidelines were introduced across the Stanford Internal Medicine resident clinics, Internal Medicine faculty clinics, and Family Medicine faculty clinics during September 2013. More than 95% of the patient encounters of interest occurred in the same physical building, with a small subset of faculty clinics operating out of a separate clinic location. Guidelines were disseminated through presentation at mandatory clinic workday meetings and e-mail distribution. The members of the opioid working group further disseminated guideline education via social marketing, based on prior evidence of the improved likelihood of behavior change.^[14]^

Specifically, the guidelines and pain agreement were reviewed for 45 minutes during a scheduled monthly clinic faculty meeting at the primary clinic location by the guideline authors. They were also presented to the residents in clinic during weekly preclinic teaching sessions for half an hour. Guidelines were disseminated in 2 separate e-mails from the clinic chiefs to all faculty and residents in the participating clinics. Guidelines were placed on a protected internal clinic website used by faculty and residents in the clinics as well as paper copies placed in a visible area of the resident teaching rooms for ongoing review. In addition, members of the working group used their personal connections with other attendings and residents to informally promote the guidelines during and after the launch period.

### 2.3 Patient population and data collection

We defined pre- and postintervention evaluation periods to identify changes in patient and provider behaviors. A run-in period from September 2013 to November 2013 was allowed for initial dissemination of the guideline content and shift in practice patterns. This yielded seasonally matched preintervention (11/1/2012–6/1/2013) and postintervention (11/1/2013–6/1/2014) evaluation periods.

We extracted electronic medical records for pre- and postintervention patient cohorts via the Stanford Translational Research Integrated Database Environment clinical data warehouse,^[18]^ as approved by the Stanford Institutional Review Board. The clinical data warehouse serves as a regularly updated copy of the Stanford hospital and clinics electronic medical records with consultation services for customized data extraction to support clinical research. All patients visiting a Stanford primary care clinic (family medicine or internal medicine) were considered. Within these cohorts, we focused on those receiving any opioid Rxs and then those receiving 3 or more opioid Rxs during the evaluation period as “chronic opioid users.” This would count 3 refills of the same medication as well as 3 separate Rxs for distinct medications. Patients with any cancer Dx in their problem list, broadly defined by ICD9 (International Classification of Diseases, Ninth Edition) codes 140 to 239, were excluded from consideration. Supplementary Table 1 includes the full list of opioid Rxs considered, along with oral morphine equivalent estimates based on active ingredients of buprenorphine, fentanyl, hydrocodone, hydromorphone, methadone, morphine, oxycodone, or oxymorphone. Notably, codeine was not counted as it is more typically prescribed here for cough suppression than for pain. To assess for balanced cohorts, we compared the preintervention and postintervention patients by baseline demographics (age, gender, and race) and prevalence of their most common ICD9 problem list diagnoses.

Provider and patient behavior outcome measures are specified below. These were selected based on their relevance to the intervention and reliability of extraction from structured electronic medical records. We compared these for pre- versus postintervention “chronic opioid users” by χ^2^ testing for categorical data and by 2-tailed *t* tests for quantitative data with a significance threshold of 0.05.

Provider behavior measures are as follows:

1. Ordered urine toxicology screen
2. Referral to physical therapy (PT)
3. Referral to psychiatry
4. Referral to pain clinic
5. Number and total morphine equivalents of opioid Rxs
6. Total percentage of clinic patients prescribed chronic opioids.

Patient behavior measures are as follows:

1. Number of primary clinic visits
2. Number of specialty referrals (pain, psychiatry, and PT) actually visited
3. Number of (Stanford) emergency department visits
4. Number of (Stanford) emergency department opioid Rxs.

## 3 Results

The Stanford primary care clinics treat a diverse group of patients throughout the San Francisco Bay Area and Northern California. They are an average of 54 years old (standard deviation 19), 45% male, 50% White, 23% Asian, and 4% Black. Overall, 31% of the clinic population has public insurance (Medicare or Medicaid), while most (63%) are privately insured. As outlined in Table 1, the primary care clinics treated 12,897 patients in the preintervention period, of whom 5995 had no cancer-related Dx. Of the noncancer patients, 234 received at least 1 opioid Rx, while 119 were counted as “chronic opioid patients” with 3 or more opioid Rxs. Of the 13,066 total patients seen by the primary care clinics in the postintervention period, 217 were noncancer patients receiving at least 1 opioid Rx, while 104 were chronic opioid patients. Note that the patients in the preintervention cohort may overlap with the postintervention cohort if they received chronic opioids both before and after the intervention. Relative to the total noncancer clinic populations considered, the above counts reflect a 14% drop in patients receiving any opioid Rx from 3.9% to 3.4% (*P* = 0.02) and a 19% drop in chronic opioid patients from 2.0% to 1.6% (*P* = 0.03).

**Table 1:**
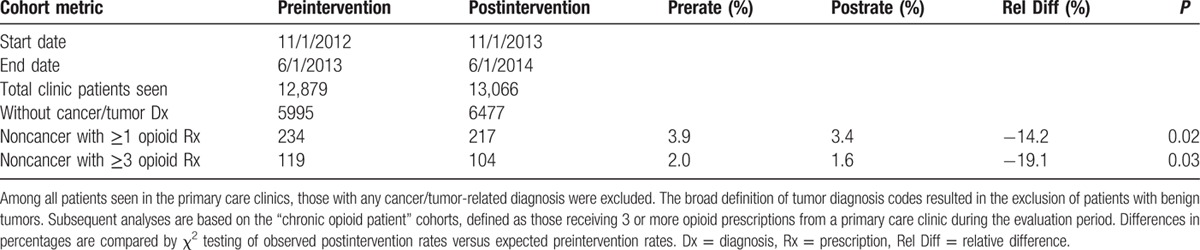
Pre- and postintervention patient cohort metrics.

Table 2 reports demographic information for the pre- and postintervention chronic opioid patient cohorts, along with the rates of the most prevalent problem list diagnoses. No significant differences in baseline demographics or comorbid diagnoses were noted between the groups.

**Table 2:**
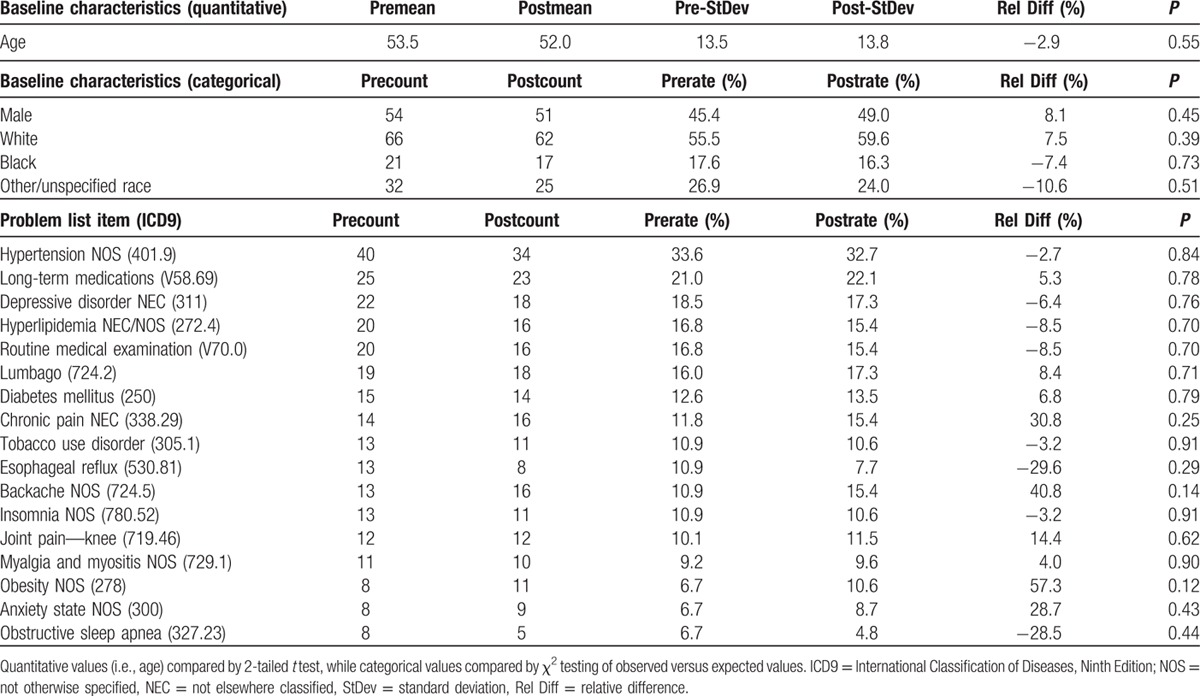
Baseline demographics and top problem list items for patients identified as “chronic opioid patients” based on 3 or more prescriptions for opioids during the pre- or postintervention periods.

Tables 3 and 4 report differences in pre- versus postintervention measures of patient and provider behavior. The percentage of chronic opioid patients subject to urine drug screening increased 87% from 9.2% to 17.3% (*P* = 0.005), but the overall rates of screening and referrals to PT, psychiatry, and pain clinic remained relatively low both pre- and postintervention. No significant differences were detected in the number of patient visits to specialty referral clinics, emergency room encounters, or the overall quantity of opioids prescribed per patient.

**Table 3:**
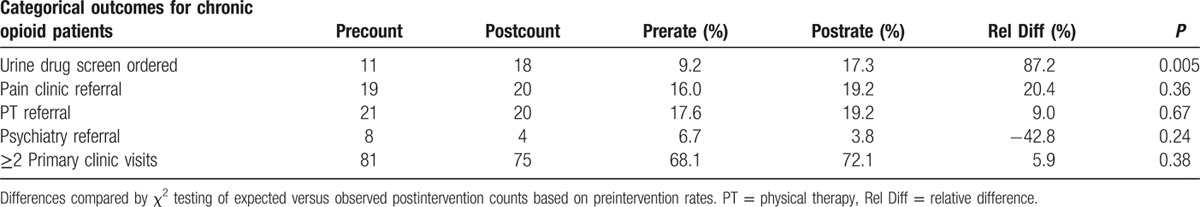
Counts of chronic opioid patients in pre- and postintervention periods for different categorical outcome measurements.

**Table 4:**
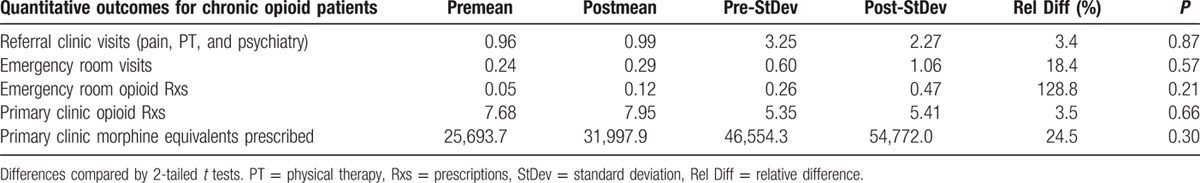
Average values and standard deviations for quantitative outcome measurements per chronic opioid patient in pre- and postintervention periods.

## 4 Discussion

Extracting structured data from patient electronic medical records allowed us to efficiently assess the effects of introducing opioid prescribing guidelines into a primary care clinic setting. This educational intervention was associated with a modest but statistically significant decrease in the percentage of clinic patients receiving any or chronic opioid Rxs. More dramatic changes could be observed in different settings with more prevalent chronic opioid use, as the baseline percentage of chronic opioid users in our primary care clinics was already relatively low (2%), compared to nationally quoted rates of from 3% to 9% depending upon the population prevalence of mental health and substance abuse problems.^[19]^

Any differences noted are unlikely to be due to shifting patient populations, as we found stable baseline demographics and top problem list diagnoses in the pre- and postintervention groups. In reviewing the most prevalent comorbid diagnoses, we note that generally common chronic conditions like hypertension and hyperlipidemia are still common amongst chronic opioid patients. Diagnoses like depression, tobacco use, backache, insomnia, obesity, and anxiety, however, appear to be more prevalent comorbidities in this population. The prevalence of lumbago (low back pain), knee joint pain, and myalgia (proxy for fibromyalgia which has no direct ICD9 code) indicates likely diagnoses for the chronic opioid Rxs, though medical documentation practices are not consistent enough to directly link diagnoses and Rx indications. While some of these patients had documented “chronic pain” and “long-term medication” in their problem list, overall structured documentation of chronic pain and opioid use was low (<25%) in both the pre- and postperiods.

The exclusion of patients with any cancer-related Dx code was extremely stringent, eliminating about half the clinic population from consideration, even though we do not observe half our patients suffering from malignant disease. In reviewing the ICD9 Dx codes, we found that many such patients were excluded for benign tumors (most commonly of the colon and skin), as in Supplementary Tables 2 and 3. Such stringent exclusion criteria reduce the power of our study to detect significant differences in the pre- and postcohorts, but helps ensure the validity of our conclusions for noncancer pain treatment.

Dissemination of the opioid guidelines was associated with a significant increase in urine toxicology screening, an important and underused monitoring tool recommended by numerous professional societies from pain and medicine to neurology.^[20]^ The absolute quantity remained relatively low however, and no obviously aberrant results were detected for those patients complying with the drug testing. Other recommended provider behavior such as specialty referrals and regular clinic visits did not significantly change. One hypothesis to explain why only the urine drug screen rate changed is that the other interventions (PT, pain, psychiatry, and primary care visits) may already have had baseline buy-in by preintervention patients as ways to help their (pain) complaints. In contrast, urine drug screening is a simple task to complete for both provider and patient, but is not directly intended to improve their symptoms.

When introducing practices that tend to deter prescribing opioids in primary care clinics, a potential unintended consequence is to drive patients to seek drugs from other sources. Within the scope of the Stanford healthcare system at least, the average number of emergency room opioid Rxs per chronic opioid patient appeared to increase from 0.05 to 0.12, but did not achieve statistical significance. This will be an important point of study for future studies powered to detect such small differences. Even if the trend holds, it would imply that about 1 in 20 chronic opioid patients received an extra emergency room opioid Rx (about 5 patients) as compared to the additional 1 in 250 clinic patients off of chronic opioids (more than 50 patients). Given data from a single health system, we cannot confirm whether there may instead have been a shift toward patients seeking opioids from outside providers (or even illicit sources). Similarly, our evaluation of structured clinical records limits the ability to assess for subjective assessments of pain, patient satisfaction, and prescriber attitudes that could have been adversely affected by limiting opioid Rxs.

**Figure 1:**
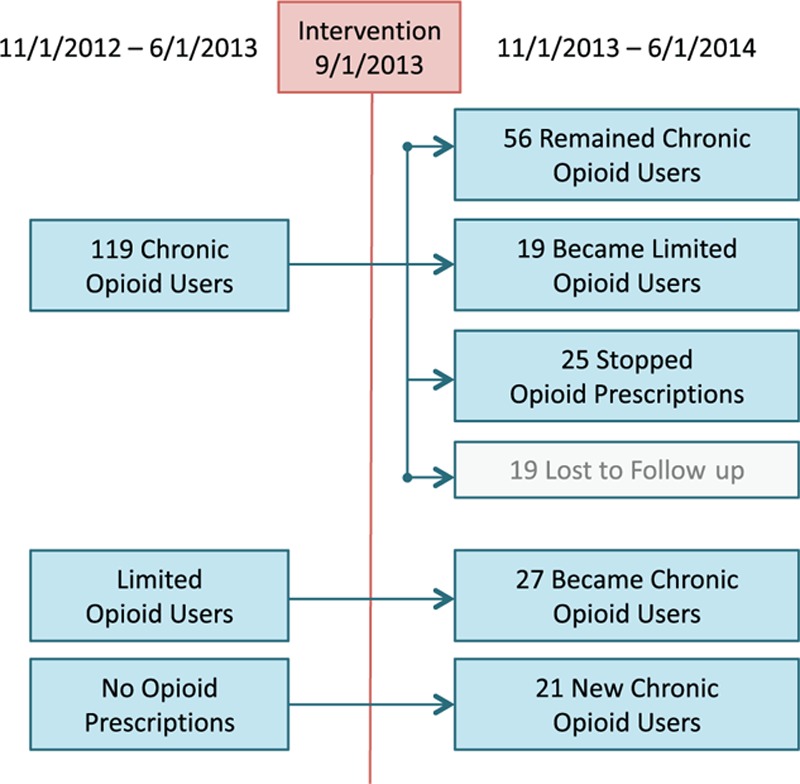
Redistribution of (noncancer) patients receiving chronic opioid prescriptions after dissemination of opioid prescribing guidelines to clinics. Chronic opioid use defined as patients receiving 3 or more opioid prescriptions within a 7-month evaluation period. Limited opioid users defined as those receiving 1 or 2 prescriptions. Stopped opioid prescriptions reflect patients with follow-up data in the postintervention period, but no opioid prescriptions. No opioid prescriptions reflect patients without opioid prescriptions in the preintervention clinical data source. During the preintervention period, 119 patients were identified as chronic opioid users, but 19 were lost to follow-up. For the 100 chronic opioid patients with follow-up data, their average number of opioid prescriptions dropped from 7.67 to 5.52 and only 56 remained chronic opioid users. At the same time, 27 limited opioid users and 21 patients not receiving any opioid prescriptions subsequently joined the total 104 chronic opioid patients in the postintervention period.

Focusing on the subgroup of chronic opioid patients in the preintervention period and following them into the postintervention period illustrates the trends in Fig. 1. The cohort received an average of 7.67 opioid Rxs in the preintervention period, down to 5.52 postintervention. Of the 119 chronic opioid users in the preintervention cohort, only 56 remained chronic opioid users in the postintervention cohort. While these differences are substantial, direct interpretation with respect to the intervention is not possible as some “regression to the mean” is expected.^[21]^ While 48 other patients joined the chronic opioid user group by the postintervention period, there was still a net decrease in the prevalence of chronic opioid users in a growing clinic population (Table 1). This remains an important reflection that prescribing guidelines are not intended to completely prevent chronic opioid use, but rather to apply a structured process to manage their relative risks and benefits.

While recognizing the limitations of a retrospective pre- and poststudy that may only detect secular trends unrelated to the intervention at hand, our primary care clinic providers have still learned valuable lessons. Overall, clinic providers found the intervention feasible to implement within their existing workflow. The primary pressure point was patient–provider time constraints, particularly for patients with limited health literacy or English proficiency. The direct patient–provider counseling necessary for discussing opioid weaning strategies, alternative methods of pain control, and related topics is predictably more time consuming than simply refilling Rxs. To manage such time constraints, our clinics are currently exploring the inclusion of pharmacists into the workflow for chronic pain management. During in-person visits and follow-up phone calls, staff pharmacists could then help counsel patients regarding nonopioid pharmacologic alternatives, assess for drug–drug interactions, and query Rx drug monitoring program databases. Clinic providers appeared to “buy in” to the intervention favorably, recognizing it as the product of an internal multidisciplinary group of peers, as opposed to nonspecific guidelines from a national organization. The inclusion of resident clinics in the intervention may diminish observed effects given that the majority of residents are only transiently affiliated with the institution, are not pursuing primary care careers, and thus have little investment into institutional education goals. That any behavioral changes could be effected through simple educational interventions at all is thus ultimately encouraging.

An educational intervention for opioid prescribing in a general primary care setting is feasible and may be expected to modestly reduce overall opioid Rx rates and increase provider use of systematic monitoring methods (i.e., urine drug screening) without unintended consequences of patients redirecting to local emergency rooms. Overall compliance with many guideline recommendations may remain low or unchanged, however, questioning the effectiveness of many emphatically promoted (but evidence-light) opioid and chronic pain management guidelines. Additional interventions like targeted population health reports,^[13]^ systems interventions through electronic clinical decision support, patient-focused strategies, financial incentives, and provider detailing^[22]^ are likely necessary to incentivize guideline implementation and systematically change provider and patient behavior in the face of this complex problem.

## Acknowledgments

We gratefully acknowledge the expertise of Dr Baldeep Singh, Dr Casey Crump, and Isabella Chu in reviewing a draft of the manuscript. Dr Chen was supported by NIEHS K01ES026837. Dr Chen and Richman were supported in part by the VA Office of Academic Affiliations and Health Services Research and Development Service Research funds.

Publication support provided by the Stanford Division of General Medical Disciplines and the Rathmann Family Foundation.

## Supplementary Material

Supplemental Digital Content
