# Supplementary material for: Effect of opioid prescribing guidelines in primary care
Source: Medicine (Baltimore). 2016 Sep 2;95(35):e4760. doi: 10.1097/MD.0000000000004760 (PMC5008612; doi:10.1097/MD.0000000000004760)
Supplement: Supplemental Digital Content [file medi-95-e4760-s001.docx]

**STANFORD PRIMARY CARE**

**OPIOID THERAPY IN CHRONIC NON-CANCER PAIN**

**Introduction**:

**Goals**:

- To provide consistent “best practice” care and universal precautions to Stanford Primary Care patients for safe and effective prescribing of opioids for chronic pain to maximize pain reduction and functional improvement.
- To prevent and reduce opioid related morbidity, mortality, dependence, diversion and addiction.

The treatment of chronic non-cancer pain (CNCP) with chronic opioid therapy remains controversial. Patients with more complex cases of chronic pain, including those with disabling pain, tend to have better outcomes when managed with a comprehensive approach that includes non-opioid modalities that address functional impairment and psychosocial factors. Recent data indicates an increase in chronic opioid use for CNCP in the United States with a dramatic increase in accidental deaths. In 2010 there were 38,329 drug overdose deaths in the U.S. 57% of these deaths involved prescription drugs and 75% involved opioids. At least 74% of these overdose deaths were unintentional. Diverted opioids account for a majority of overdose deaths in adolescents and young adults. Prescription opioids currently lead to more unintentional overdose deaths than cocaine and heroin combined.

Compliance with all applicable California/Federal law/regulation is met prior to initiating treatment for chronic, non-cancer pain with opioid pain medication.

**When to consider opioid therapy for chronic pain:**

- Chronic opioid therapy may be considered after a detailed history, physical exam and appropriate testing including the assessment of risk or current substance abuse, misuse or addiction.
- A detailed history is obtained of attempts, duration, effectiveness, and any adverse effects of physical therapy, cognitive behavioral therapy, NSAID’s, antidepressants, antiepileptics, non-opioid analgesics and local therapies such as topical lidocaine.
- When other physical, behavioral and non-opioid measures have failed to adequately control moderate to severe pain.
- Abnormal sleep patterns have been addressed.
- Co-morbid medical issues that could increase risk of opioid related overdose have been addressed (e.g. URI, influenza, COPD, pneumonia).
- The patient has demonstrated sustained improvement in function and pain levels in previous opioid trials.
- The benefits and risks of opioid use are carefully weighed using risk assessment tools such as the Opioid Risk Tool. (see appendix)
- The patient understands the ongoing goal of opioid taper and discontinuation as other modalities are utilized in the treatment plan.
- A state drug monitoring program such as California’s Controlled Substance Utilization Review and Evaluation System (C.U.R.E.S.) has been queried.
- The patient is able and willing to return for ongoing regular care at Stanford Primary Care.

**Guidelines for Initial Evaluation of Patients with Chronic Pain or New Patients to the Clinic:**

- Obtain the following *prior to intiating chronic opioid treatment* or when evaluating new patients to the clinic who are already on opioid treatment:
  - Clear diagnosis for cause of pain and/or an appropriate differential diagnosis in Problem List (consider with supporting labs, imaging, consults, etc).
  - Patient’s outside records regarding evaluation and treatment of chronic pain.
  - CURES report.
  - Baseline functional assessment.
  - Use or abuse of alcohol, illicit drugs, other scheduled medications, benzodiazepines, barbiturates and other sedative hypnotics.
  - Use of non opioid treatment modalities, particularly any current medications that might negatively interact with opioids.
  - Psychiatric history, personal or family history of substance abuse, history of preadolescent sexual abuse (use Opioid Risk Tool, available in appendix).
  - Patient should complete a brief depression inventory (consider PHQ2/PHQ9, DAST, or Beck Depression Inventory) and screen for anxiety and PTSD.
  - Assessment of social factors that might impact pain management including: employment, job satisfaction, marital history and history of legal problems.
  - Baseline urine drug screen Review of past medical records re: pain diagnosis
  - Incorporate new REMS guidelines for long-acting opiates.

*A physician may at his/her discretion opt not to prescribe opioids prior to obtaining the patient’s outside records.*

- When instituting chronic opioid therapy, both prescriber and patient should:
- Review and sign the Stanford Primary Care Pain Agreement. The agreement must be scanned in EPIC. Place the phrase “PAIN MEDICATION AGREEMENT” with the name of the PMD who signed the contract and the date signed in the problem list. Code ICD Pain Management Contract Signed (V58.69K).

**Monitoring Patients on Chronic Opioids:**

- Patients should be routinely monitored on therapy and a follow up plan should be established with the patient at each visit.
- Patients should be seen in clinic *at least once every 3 months* while on chronic opioids.
- Assess both functional improvement and pain relief while on opioid therapy.
- Communicate with patients that opioid therapy is a trial and is part of a comprehensive pain treatment plan that requires ongoing evaluation.

***Monitor* and *Document* the following while patients are on chronic opioids:**

- Assess the “4-A’s,” which are *analgesia, activity, adverse effects and aberrant behaviors.*
- Consider use of Chart Tools/Scales to monitor patient’s functional status, pain level, adverse effects and aberrant behaviors (see appendix).
- The PEG scale (Pain, Enjoyment, General Activity) scale is a *brief* pain measure that patients can self-complete and is comprised of three questions to assess the patient’s pain levels and activity.
- Functional scales help to assess for functional improvement. Consider using the PROMIS Physical Function Short Form (see appendix).
- Longer validated scales to assess the “4-A’s” may be found in the appendix.
- Be aware that pain interference scales may not identify functional status changes.
- Monitor adherence to prescribed therapies (dosage, frequency) and document other non-opioid treatment modalities.
- Monitor and document *adverse effects*: nausea, vomiting, constipation, itching, mental cloudiness, sweating, fatigue, urinary retention and drowsiness.
- Continue to monitor and document the patient’s use of alcohol, illicit drugs, other controlled medications, sedatives and psychiatric history.
- Obtain a CURES report on the patient at least annually.
- *Aberrant Behaviors* to identify:
  - Negative mood changes, deterioration in functioning at work or socially, lost or stolen prescriptions, resistance to change therapy despite adverse effects, refusal to comply with random drug screens, concurrent use of illicit drugs or alcohol, use of multiple physicians or pharmacies, illegal activities, requests for frequent renewals, use of pain medication in response to situational stressors, insisting on certain medications by name, hoarding medication, unsanctioned dose escalation and non-adherence to other recommendations for pain treatment.
- Be aware of the differential diagnosis of aberrant behavior, including: inadequate analgesia (pseudo-addiction), disease progression, opioid resistant pain, addiction, opioid analgesia tolerance and diversion.
- Random and periodic urine drug testing should be used as part of the monitoring process.
- Consider random pill counts and ask the patient to bring the pill bottles to each appointment.
- Continue to assess the benefits/harms of opioid treatment and communicate with the patient the goals of treatment. Set realistic expectations that pain will likely not be eliminated. Emphasize that the goal of treatment is to reduce pain and increase functionality.
- All opioid prescriptions should be added into EPIC as medication orders with the “no print” function with the correct number of pills prescribed, as well as refills, if applicable. Stable patients can be issued 3 months (dated when signed but with DO NOT FILL for the 2^nd^ and 3^rd^ Rx months) based on clinical discretion.
- All scheduled medications written on secure scripts must also be documented as a prescription in EPIC.
- The medical diagnoses related to the patient’s chronic pain should be updated and placed in the Problem List.
- Remind patients to lock up opioids to prevent diversion.

**Principles on Opioid Dosing for Chronic Non-cancer Pain (CNCP):**

- The total daily dose of opioids should not be increased above 100 mg oral morphine equivalent dose (MED) without either the patient demonstrating improvement in function and pain or first obtaining a consultation from a practitioner qualified in chronic pain management. Data supports that the risks of opioid related adverse events increases with dose.
- Opioid conversion calculators are estimates of equianalgesic doses and caution should be taken when conversions are used.

| **Opioid** | **Approximate Equianalgesic Dose (oral & transdermal)** |
| --- | --- |
| **Morphine (reference)** | **30 mg** |
| Codeine | 200 mg |
| Fentanyl transdermal | 12.5 mcg/hr |
| Hydrocodone | 30 mg |
| Hydromorphone | 7.5 mg |
| Methadone | 7.5 mg |
| Oxycodone | 20 mg |
| Oxymorphone | 10 mg |
| Tapentadol | 81 mg |
| Tramadol | 150 mg |

**Principles for safely prescribing chronic opioid therapy:**

- Single prescriber.
- Single pharmacy.
- The patient and prescriber have a signed PAIN AGREEMENT.
- The lowest possible effective dose should be used.
- Be cautious when using opioids with conditions that may potentiate opioid adverse effects (including COPD, CHF, sleep apnea, current or past alcohol or substance abuse, elderly or history of renal or hepatic dysfunction).
- Do not combine opioids with sedative-hypnotics, benzodiazepines, muscle relaxants, or barbiturates for chronic non-cancer pain unless there is a specific medical and/or psychiatric indication for the combination.
- Exercise caution with opioid-acetaminophen combination drugs to ensure daily acetaminophen dose not exceed 4.0 grams.
- Covering PCP may honor refill requests for opioid pain medication if :
  - Pain medication agreement is in record.
  - No violation of agreement apparent.
  - Refill due date is clearly documented.
  - May choose to order urine drug screen.
  - May deny refill for cause.

**Urine testing:**

- A baseline urine drug test (UDT) should be performed on all transferring patients who are already using opioids and for those patients who you are considering for chronic opioid therapy. Prior to testing, the prescriber should inform the patient of the reason for testing, the expectation of random repeat testing and consequences of unexpected results. This gives the patient an opportunity to disclose drug use and allows the prescriber to modify drug testing for the individual circumstances and more accurately interpret the results.

**Red Flags for Urine Testing:**

- Negative for opioid(s) you prescribed.
- Positive for amphetamine or methamphetamine.
- Positive for cocaine or metabolites.
- Positive for drug (benzodiazepines, opioids, etc.) you did not prescribe.
- Positive for alcohol.

**EPIC ordering:**

- **Drugs of Abuse Screen, Urine (approximately $12):**
- Detects the following: Amphetamines, Barbiturates, Benzodiazepines, Cocaine, Methadone, other Opiates, PCP, THC, Tricyclics.
- Does not detect fentanyl.
- Does not differentiate between different opiates (i.e., oxycodone and hydromorphone) other than methadone.
- Test results are non-quantitative (i.e., positive or negative).
- False positives are possible on the opioid screen with ofloxacin or levofloxacin.
- False positives are possible on the amphetamines screen with some cold medicine.
- **Confirmed Drug Abuse Survey, Urine (approximately $12 for initial screen, if part two is needed, then part two may cost approximately $200):**
- Detects the following: Ethanol, Amphetamines, Barbiturates, Benzodiazepines, Cocaine, Opiates, PCP, THC.
- Two-part test (first part is an antibody-based screening test, second part is a confirmatory gas chromatography/mass spectroscopy test).
- Provides quantitative results for a particular drug if a patient screens positive.
- Identifies specific drugs if a patient screens positive (i.e., if a patient screens positive for amphetamines, the second part of this test separately looks for and quantifies amphetamines, methamphetamines, and pseudoephedrine, eliminating the possibility of cold medicine-related false positive testing; if a patient screens positive for opioids, the test will identify and quantify each specific opioid, allowing potential detection of opioids that you haven’t prescribed in addition to those that you have prescribed).
- Initial screening test is relatively inexpensive. If confirmatory testing is required, higher costs are incurred.
- Test is currently a send-out to Mayo and results take several days to come back; an in-house version of this test may be available as soon as July 2013.

**Contact/Agreement Violations:**

- Provider should chart every violation of agreement and document response in chart.
- If one major violation of the agreement occurs (falsifying, diversion, selling, confirmed lab evidence of substance abuse, refusal to comply with urine testing, etc.), this will lead to discontinuation of prescribing opiates and patients should be counseled or referred for treatment.
- If two minor violations of the agreement occur (attempting to obtain the opiate from another MD, claims of lost or stolen prescriptions, early refill requests, missing appointments), then consider modification of agreement at a minimum.

**Tapering or discontinuing opioids:**

Not all patients benefit from opioids, and a prescriber frequently faces the challenge of reducing the opioid dose or discontinuing the opioid altogether. Weaning from opioids can be done safely by slowly tapering the opioid dose and taking into account the following issues:

- A decrease by 10% of the original dose per week is usually well tolerated with minimal physiological adverse effects. Some patients can be tapered more rapidly without problems (over 6 to 8 weeks). Tramadol can be decreased by 25% every 1 – 2 weeks.
- Opioid withdrawal is rarely medically serious although symptoms may be unpleasant.
- Symptoms of an abstinence syndrome, such as nausea, diarrhea, muscle pain and myoclonus can be managed with clonidine 0.1 – 0.2 mg orally every 6 hours or clonidine transdermal patch 0.1 mg/24hrs weekly during the taper while monitoring often for significant hypotension and anticholinergic side effects. Zofran can also reduce withdrawal symptoms due to its effects on 5HT3 (unrelated to its antiemetic features). In some patients it may be necessary to slow the taper timeline to monthly rather than weekly dosage adjustments.
- Other comfort medications that may be used include: dicyclomine 20 mg every 6 hours for stomach cramping, antiemetics, antidiarrheals (i.e., loperamide, bismuth) and decongestants.
- Symptoms of mild opioid withdrawal may persist for six months after opioids have been discontinued. Rapid reoccurrence of tolerance can occur for months to years after prior chronic use.
- Consider using adjuvant agents such as antidepressants to manage irritability or sleep disturbance, or antiepileptics for neuropathic pain.
- Emphasize that tolerance can be lost within a few days and often causes accidental over dose. Long-term opiate abstinence symptoms sometimes do not resolve because the endogenous opiate system does not normalize and long-term opiates may be necessary.

**Reasons for Referral to Pain Management:**

Consultative assistance for opioid management and prudent prescribing of opioids should be sought with a pain management expert under the following conditions:

- To aid with a complex pain condition or if there is a need for help with a diagnosis or verification of a diagnosis.
- To assist with a patient with significant co-morbidities.
- The clinician suspects development of significant tolerance to opioids.
- To assist with further assistance with assessment of risk/benefit of chronic opioid therapy.
- Patients on > 100 mg MED/day.
- Difficulty with tapering patients off opioids.
- Aberrant behavior.
- Adjunctive treatments are being considered (i.e., epidurals, etc.).
- Any patient on opioid medications longer than a year. (Many suggest regular yearly reassessment by pain consultants if a patient continues to need chronic opioids).

Consultation with a specialist does not necessitate transfer of the patient for care or ongoing opioid prescribing. However, the consultant should advise the prescribing provider on a pain management plan and may include: alternative treatments to reduce or discontinue use of opioids, explanation of the risks and benefits of a possible trial with opioids above 120 mg/day MED and the need for ongoing documentation of improvement in function and pain. Establish ongoing communication with the pain specialist to ensure that the treatment plan is clearly outlined for the patient and the provider responsible for prescribing medications is well-defined.

**Supplementary Table 1 -** Oral morphine equivalents assigned to different medications

| **Oral Morphine Equiv** | **Pre- Rxs** | **Pre- Patients** | **Post- Rxs** | **Post- Patients** | **Medication Description** |
| --- | --- | --- | --- | --- | --- |
| **150** | . | . | . | . | BUPRENORPHINE 2 MG SL SUBL |
| **600** | . | . | . | . | BUPRENORPHINE 8 MG SL SUBL |
| **600** | . | . | . | . | BUPRENORPHINE-NALOXONE 8-2 MG SL SUBL |
| **16** | . | . | . | . | DILAUDID 4 MG PO TABS |
| **2160** | 12 | . | . | . | FENTANYL 100 MCG/HR TD PT72 |
| **259.2** | . | . | . | . | FENTANYL 12 MCG/HR TD PT72 |
| **540** | . | . | 14 | . | FENTANYL 25 MCG/HR TD PT72 |
| **1080** | 14 | . | 17 | . | FENTANYL 50 MCG/HR TD PT72 |
| **1620** | . | . | 13 | . | FENTANYL 75 MCG/HR TD PT72 |
| **10** | 106 | 31 | 117 | 41 | HYDROCODONE-ACETAMINOPHEN 10-325 MG PO TABS |
| **10** | . | . | . | . | HYDROCODONE-ACETAMINOPHEN 10-500 MG PO TABS |
| **0.5** | . | . | . | . | HYDROCODONE-ACETAMINOPHEN 2.5-108 MG/5 ML PO SOLN |
| **5** | 34 | 13 | 170 | 92 | HYDROCODONE-ACETAMINOPHEN 5-325 MG PO TABS |
| **5** | 303 | 109 | 91 | 49 | HYDROCODONE-ACETAMINOPHEN 5-500 MG PO TABS |
| **7.5** | 26 | . | 29 | . | HYDROCODONE-ACETAMINOPHEN 7.5-325 MG PO TABS |
| **7.5** | . | . | . | . | HYDROCODONE-ACETAMINOPHEN 7.5-500 MG PO TABS |
| **0.5** | . | . | . | . | HYDROCODONE-ACETAMINOPHEN 7.5-500 MG/15 ML PO SOLN |
| **0.5** | . | . | . | . | HYDROCODONE-ACETAMINOPHEN 7.5-500 MG/15 ML(15 ML) PO SOLN |
| **7.5** | 16 | . | . | . | HYDROCODONE-ACETAMINOPHEN 7.5-750 MG PO TABS |
| **0.3** | . | . | . | . | HYDROCODONE-HOMATROPINE 5-1.5 MG/5 ML PO SYRP |
| **7.5** | 24 | . | 20 | . | HYDROCODONE-IBUPROFEN 7.5-200 MG PO TABS |
| **8** | . | . | 15 | . | HYDROMORPHONE 2 MG PO TABS |
| **16** | . | . | 10 | . | HYDROMORPHONE 4 MG PO TABS |
| **32** | . | . | . | . | HYDROMORPHONE 8 MG PO TABS |
| **10** | . | . | . | . | KADIAN 10 MG PO CSRP |
| **10** | . | . | . | . | LORTAB 10-500 MG PO TABS |
| **7.5** | . | . | . | . | LORTAB 7.5-500 MG PO TABS |
| **0.5** | . | . | . | . | LORTAB ELIXIR 7.5-500 MG/15 ML PO SOLN |
| **80** | 65 | 12 | 104 | 14 | METHADONE 10 MG PO TABS |
| **16** | . | . | . | . | METHADONE 10 MG/5 ML PO SOLN |
| **80** | . | . | . | . | METHADONE 10 MG/ML PO CONC |
| **40** | 31 | 10 | 33 | . | METHADONE 5 MG PO TABS |
| **8** | . | . | . | . | METHADONE 5 MG/5 ML PO SOLN |
| **10** | . | . | . | . | MORPHINE 10 MG PO TAB |
| **2** | . | . | . | . | MORPHINE 10 MG/5 ML PO SOLN |
| **100** | 15 | . | 13 | . | MORPHINE 100 MG PO TBSR |
| **15** | . | . | . | . | MORPHINE 15 MG PO CAPS |
| **15** | . | . | . | . | MORPHINE 15 MG PO TABS |
| **15** | 29 | . | 29 | 10 | MORPHINE 15 MG PO TBSR |
| **15** | 29 | . | 29 | 10 | MORPHINE 15 MG PO TBSR |
| **4** | . | . | . | . | MORPHINE 20 MG/5 ML PO SOLN |
| **30** | . | . | . | . | MORPHINE 30 MG PO TAB |
| **30** | 14 | . | 15 | . | MORPHINE 30 MG PO TABS |
| **30** | 12 | . | . | . | MORPHINE 30 MG PO TBSR |
| **60** | 21 | . | . | . | MORPHINE 60 MG PO TBSR |
| **20** | . | . | . | . | MORPHINE CONCENTRATE 100 MG/5 ML (20 MG/ML) PO SOLN |
| **15** | . | . | . | . | MS CONTIN 15 MG PO TBSR |
| **30** | 14 | . | . | . | MS CONTIN 30 MG PO TBSR |
| **60** | . | . | . | . | MS CONTIN 60 MG PO TBSR |
| **10** | 18 | . | 44 | 15 | NORCO 10-325 MG PO TABS |
| **5** | . | . | . | . | NORCO 5-325 MG PO TABS |
| **7.5** | . | . | . | . | NORCO 7.5-325 MG PO TABS |
| **15** | 45 | 10 | 34 | 10 | OXYCODONE 10 MG PO TABS |
| **15** | 17 | . | 12 | . | OXYCODONE 10 MG PO TB12 |
| **22.5** | 31 | . | 32 | . | OXYCODONE 15 MG PO TABS |
| **22.5** | . | . | . | . | OXYCODONE 15 MG PO TB12 |
| **30** | . | . | . | . | OXYCODONE 20 MG PO TABS |
| **30** | 17 | . | 15 | . | OXYCODONE 20 MG PO TB12 |
| **45** | 17 | . | 23 | . | OXYCODONE 30 MG PO TABS |
| **45** | . | . | . | . | OXYCODONE 30 MG PO TB12 |
| **60** | 13 | . | . | . | OXYCODONE 40 MG PO TB12 |
| **7.5** | . | . | . | . | OXYCODONE 5 MG PO CAPS |
| **7.5** | 45 | 15 | 57 | 19 | OXYCODONE 5 MG PO TABS |
| **1.5** | . | . | . | . | OXYCODONE 5 MG/5 ML PO SOLN |
| **90** | . | . | . | . | OXYCODONE 60 MG PO TB12 |
| **120** | . | . | . | . | OXYCODONE 80 MG PO TB12 |
| **7.5** | . | . | . | . | OXYCODONE HCL-OXYCODONE-ASA 4.5-0.38-325 MG PO TABS |
| **15** | 22 | . | 32 | 10 | OXYCODONE-ACETAMINOPHEN 10-325 MG PO TABS |
| **15** | . | . | . | . | OXYCODONE-ACETAMINOPHEN 10-650 MG PO TABS |
| **7.5** | 36 | 14 | 15 | 11 | OXYCODONE-ACETAMINOPHEN 5-325 MG PO TABS |
| **7.5** | . | . | . | . | OXYCODONE-ACETAMINOPHEN 5-325 MG/5 ML PO SOLN |
| **11.25** | . | . | . | . | OXYCODONE-ACETAMINOPHEN 7.5-325 MG PO TABS |
| **15** | . | . | 19 | . | OXYCONTIN 10 MG PO TB12 |
| **30** | . | . | . | . | OXYCONTIN 20 MG PO TB12 |
| **45** | . | . | . | . | OXYCONTIN 30 MG PO TB12 |
| **60** | 14 | . | . | . | OXYCONTIN 40 MG PO TB12 |
| **90** | 17 | . | 15 | . | OXYCONTIN 60 MG PO TB12 |
| **120** | 11 | . | . | . | OXYCONTIN 80 MG PO TB12 |
| **30** | . | . | . | . | OXYMORPHONE 10 MG PO TB12 |
| **15** | . | . | . | . | OXYMORPHONE 5 MG PO TB12 |
| **15** | 29 | . | 27 | . | PERCOCET 10-325 MG PO TABS |
| **7.5** | . | . | . | . | PERCOCET 5-325 MG PO TABS |
| **11.25** | . | . | . | . | PERCOCET 7.5-325 MG PO TABS |
| **22.5** | . | . | . | . | ROXICODONE 15 MG PO TABS |
| **7.5** | . | . | . | . | ROXICODONE 5 MG PO TABS |
| **5** | 35 | 18 | 19 | 14 | VICODIN 5-500 MG PO TABS |
| **7.5** | . | . | . | . | VICODIN ES 7.5-750 MG PO TABS |
| **10** | 10 | . | . | . | VICODIN HP 10-660 MG PO TABS |

Supplementary Table 1 – Oral morphine equivalents assigned to different medication units prescribed in primary care clinics to calculate total morphine equivalents prescribed. Counts of the number of prescriptions and number of patients prescribed each in the pre- and post- intervention periods included if >= 10 (smaller counts, including 0, omitted to protect patient deidentification). Rx: Prescription, Equiv: Equivalents.

Supplementary Table 2 – “Cancer” Problem List Items from Post-Intervention Period

| **ICD9** | **Description** | **Patients** |
| --- | --- | --- |
| **211.3** | Benign neoplasm of colon | 378 |
| **174.9** | Malignant neoplasm of breast (female), unspecified | 354 |
| **185** | Malignant neoplasm of prostate | 195 |
| **216.9** | Benign neoplasm of skin, site unspecified | 180 |
| **229.9** | Benign neoplasm of unspecified site | 170 |
| **218.9** | Leiomyoma of uterus, unspecified | 153 |
| **173.91** | Basal cell carcinoma of skin, site unspecified | 103 |
| **193** | Malignant neoplasm of thyroid gland | 91 |
| **224.6** | Benign neoplasm of choroid | 89 |
| **233.0** | Carcinoma in situ of breast | 74 |
| **199.1** | Other malignant neoplasm without specification of site | 70 |
| **153.9** | Malignant neoplasm of colon, unspecified site | 60 |
| **225.2** | Benign neoplasm of cerebral meninges | 60 |
| **189.0** | Malignant neoplasm of kidney, except pelvis | 59 |
| **202.80** | Other malignant lymphomas, unspecified site, extranodal and solid organ sites | 58 |
| **214.9** | Lipoma, unspecified site | 48 |
| **188.9** | Malignant neoplasm of bladder, part unspecified | 45 |
| **215.9** | Other benign neoplasm of connective and other soft tissue, site unspecified | 43 |
| **217** | Benign neoplasm of breast | 39 |
| **227.3** | Benign neoplasm of pituitary gland and craniopharyngeal duct | 39 |
| **173.31** | Basal cell carcinoma of skin of other and unspecified parts of face | 38 |
| **216.5** | Benign neoplasm of skin of trunk, except scrotum | 37 |
| **183.0** | Malignant neoplasm of ovary | 34 |
| **162.9** | Malignant neoplasm of bronchus and lung, unspecified | 33 |
| **172.9** | Melanoma of skin, site unspecified | 33 |
| **239.0** | Neoplasm of unspecified nature of digestive system | 29 |
| **227.0** | Benign neoplasm of adrenal gland | 28 |
| **238.2** | Neoplasm of uncertain behavior of skin | 28 |
| **155.0** | Malignant neoplasm of liver, primary | 27 |
| **182.0** | Malignant neoplasm of corpus uteri, except isthmus | 27 |
| **204.10** | Chronic lymphoid leukemia, without mention of having achieved remission | 27 |
| **201.90** | Hodgkin's disease, unspecified type, unspecified site, extranodal and solid organ sites | 26 |
| **198.5** | Secondary malignant neoplasm of bone and bone marrow | 24 |
| **225.1** | Benign neoplasm of cranial nerves | 24 |
| **154.1** | Malignant neoplasm of rectum | 22 |
| **228.00** | Hemangioma of unspecified site | 22 |
| **239.2** | Neoplasm of unspecified nature of bone, soft tissue, and skin | 22 |
| **203.00** | Multiple myeloma, without mention of having achieved remission | 21 |
| **214.1** | Lipoma of other skin and subcutaneous tissue | 21 |
| **186.9** | Malignant neoplasm of other and unspecified testis | 20 |
| **223.0** | Benign neoplasm of kidney, except pelvis | 20 |
| **180.9** | Malignant neoplasm of cervix uteri, unspecified site | 19 |
| **238.4** | Polycythemia vera | 19 |
| **172.5** | Malignant melanoma of skin of trunk, except scrotum | 18 |
| **173.90** | Unspecified malignant neoplasm of skin, site unspecified | 18 |
| **216.7** | Benign neoplasm of skin of lower limb, including hip | 18 |
| **179** | Malignant neoplasm of uterus, part unspecified | 15 |
| **202.00** | Nodular lymphoma, unspecified site, extranodal and solid organ sites | 15 |
| **205.10** | Chronic myeloid leukemia, without mention of having achieved remission | 14 |
| **238.71** | Essential thrombocythemia | 14 |
| **171.9** | Malignant neoplasm of connective and other soft tissue, site unspecified | 13 |
| **209.60** | Benign carcinoid tumor of unknown primary site | 13 |
| **238.75** | Myelodysplastic syndrome, unspecified | 13 |
| **239.7** | Neoplasm of unspecified nature of endocrine glands and other parts of nervous system | 13 |
| **202.10** | Mycosis fungoides, unspecified site, extranodal and solid organ sites | 12 |
| **214.8** | Lipoma of other specified sites | 12 |
| **173.92** | Squamous cell carcinoma of skin, site unspecified | 11 |
| **216.6** | Benign neoplasm of skin of upper limb, including shoulder | 11 |
| **218.1** | Intramural leiomyoma of uterus | 11 |
| **228.04** | Hemangioma of intra-abdominal structures | 11 |
| **157.9** | Malignant neoplasm of pancreas, part unspecified | 10 |
| **170.9** | Malignant neoplasm of bone and articular cartilage, site unspecified | 10 |
| **172.6** | Malignant melanoma of skin of upper limb, including shoulder | 10 |
| **172.7** | Malignant melanoma of skin of lower limb, including hip | 10 |

Supplementary Table 2 – ICD9 codes and descriptions of patient “cancer” problem list items that resulted in exclusion of at least 10 patients from the primary study cohort. Counts reflect the number of unique patients in the post-intervention time period that were excluded due to the respective item. This included 2,844 total patients (note that some patients have multiple “cancer” problem list items). ICD9: International Classification of Diseases, Ninth Edition.

Supplementary Table 3 – “Cancer” Encounter Diagnoses from Post-Intervention Period

| **ICD9** | **Description** | **Patients** |
| --- | --- | --- |
| **216.9** | Benign neoplasm of skin, site unspecified | 1788 |
| **238.2** | Neoplasm of uncertain behavior of skin | 724 |
| **211.3** | Benign neoplasm of colon | 648 |
| **239.2** | Neoplasm of unspecified nature of bone, soft tissue, and skin | 640 |
| **174.9** | Malignant neoplasm of breast (female), unspecified | 425 |
| **229.9** | Benign neoplasm of unspecified site | 306 |
| **218.9** | Leiomyoma of uterus, unspecified | 296 |
| **228.00** | Hemangioma of unspecified site | 284 |
| **173.91** | Basal cell carcinoma of skin, site unspecified | 260 |
| **185** | Malignant neoplasm of prostate | 239 |
| **216.5** | Benign neoplasm of skin of trunk, except scrotum | 223 |
| **214.9** | Lipoma, unspecified site | 203 |
| **199.1** | Other malignant neoplasm without specification of site | 168 |
| **238.9** | Neoplasm of uncertain behavior, site unspecified | 139 |
| **173.31** | Basal cell carcinoma of skin of other and unspecified parts of face | 133 |
| **215.9** | Other benign neoplasm of connective and other soft tissue, site unspecified | 126 |
| **214.1** | Lipoma of other skin and subcutaneous tissue | 121 |
| **224.6** | Benign neoplasm of choroid | 113 |
| **233.0** | Carcinoma in situ of breast | 113 |
| **202.80** | Other malignant lymphomas, unspecified site, extranodal and solid organ sites | 99 |
| **193** | Malignant neoplasm of thyroid gland | 97 |
| **217** | Benign neoplasm of breast | 97 |
| **216.3** | Benign neoplasm of skin of other and unspecified parts of face | 85 |
| **153.9** | Malignant neoplasm of colon, unspecified site | 80 |
| **225.2** | Benign neoplasm of cerebral meninges | 75 |
| **189.0** | Malignant neoplasm of kidney, except pelvis | 73 |
| **239.0** | Neoplasm of unspecified nature of digestive system | 73 |
| **173.92** | Squamous cell carcinoma of skin, site unspecified | 70 |
| **218.1** | Intramural leiomyoma of uterus | 67 |
| **216.7** | Benign neoplasm of skin of lower limb, including hip | 66 |
| **173.3** | Other and unspecified malignant neoplasm of skin of other and unspecified parts of face | 60 |
| **172.9** | Melanoma of skin, site unspecified | 58 |
| **188.9** | Malignant neoplasm of bladder, part unspecified | 58 |
| **162.9** | Malignant neoplasm of bronchus and lung, unspecified | 54 |
| **228.01** | Hemangioma of skin and subcutaneous tissue | 53 |
| **216.1** | Benign neoplasm of eyelid, including canthus | 52 |
| **227.3** | Benign neoplasm of pituitary gland and craniopharyngeal duct | 52 |
| **216.6** | Benign neoplasm of skin of upper limb, including shoulder | 47 |
| **182.0** | Malignant neoplasm of corpus uteri, except isthmus | 41 |
| **227.0** | Benign neoplasm of adrenal gland | 41 |
| **173.90** | Unspecified malignant neoplasm of skin, site unspecified | 40 |
| **173.51** | Basal cell carcinoma of skin of trunk, except scrotum | 39 |
| **234.9** | Carcinoma in situ, site unspecified | 39 |
| **198.5** | Secondary malignant neoplasm of bone and bone marrow | 38 |
| **204.10** | Chronic lymphoid leukemia, without mention of having achieved remission | 38 |
| **155.0** | Malignant neoplasm of liver, primary | 37 |
| **238.71** | Essential thrombocythemia | 37 |
| **173.32** | Squamous cell carcinoma of skin of other and unspecified parts of face | 36 |
| **183.0** | Malignant neoplasm of ovary | 36 |
| **154.1** | Malignant neoplasm of rectum | 34 |
| **215.2** | Other benign neoplasm of connective and other soft tissue of upper limb, including shoulder | 32 |
| **172.5** | Malignant melanoma of skin of trunk, except scrotum | 31 |
| **201.90** | Hodgkin's disease, unspecified type, unspecified site, extranodal and solid organ sites | 31 |
| **238.4** | Polycythemia vera | 31 |
| **203.00** | Multiple myeloma, without mention of having achieved remission | 30 |
| **214.8** | Lipoma of other specified sites | 29 |
| **225.1** | Benign neoplasm of cranial nerves | 29 |
| **179** | Malignant neoplasm of uterus, part unspecified | 28 |
| **173.41** | Basal cell carcinoma of scalp and skin of neck | 27 |
| **173.61** | Basal cell carcinoma of skin of upper limb, including shoulder | 25 |
| **216.4** | Benign neoplasm of scalp and skin of neck | 25 |
| **218.0** | Submucous leiomyoma of uterus | 25 |
| **232.9** | Carcinoma in situ of skin, site unspecified | 25 |
| **238.75** | Myelodysplastic syndrome, unspecified | 25 |
| **209.60** | Benign carcinoid tumor of unknown primary site | 24 |
| **180.9** | Malignant neoplasm of cervix uteri, unspecified site | 23 |
| **223.0** | Benign neoplasm of kidney, except pelvis | 23 |
| **239.7** | Neoplasm of unspecified nature of endocrine glands and other parts of nervous system | 23 |
| **239.9** | Neoplasm of unspecified nature, site unspecified | 23 |
| **171.9** | Malignant neoplasm of connective and other soft tissue, site unspecified | 22 |
| **173.62** | Squamous cell carcinoma of skin of upper limb, including shoulder | 22 |
| **186.9** | Malignant neoplasm of other and unspecified testis | 22 |
| **174.8** | Malignant neoplasm of other specified sites of female breast | 21 |
| **211.1** | Benign neoplasm of stomach | 21 |
| **172.6** | Malignant melanoma of skin of upper limb, including shoulder | 20 |
| **202.00** | Nodular lymphoma, unspecified site, extranodal and solid organ sites | 20 |
| **205.10** | Chronic myeloid leukemia, without mention of having achieved remission | 20 |
| **238.79** | Other lymphatic and hematopoietic tissues | 20 |
| **173.42** | Squamous cell carcinoma of scalp and skin of neck | 19 |
| **157.9** | Malignant neoplasm of pancreas, part unspecified | 18 |
| **228.04** | Hemangioma of intra-abdominal structures | 18 |
| **239.4** | Neoplasm of unspecified nature of bladder | 18 |
| **172.7** | Malignant melanoma of skin of lower limb, including hip | 17 |
| **197.0** | Secondary malignant neoplasm of lung | 17 |
| **197.7** | Malignant neoplasm of liver, secondary | 17 |
| **233.1** | Carcinoma in situ of cervix uteri | 17 |
| **239.5** | Neoplasm of unspecified nature of other genitourinary organs | 17 |
| **239.6** | Neoplasm of unspecified nature of brain | 17 |
| **173.11** | Basal cell carcinoma of eyelid, including canthus | 16 |
| **173.21** | Basal cell carcinoma of skin of ear and external auditory canal | 16 |
| **173.72** | Squamous cell carcinoma of skin of lower limb, including hip | 16 |
| **213.9** | Benign neoplasm of bone and articular cartilage, site unspecified | 16 |
| **220** | Benign neoplasm of ovary | 16 |
| **173.71** | Basal cell carcinoma of skin of lower limb, including hip | 15 |
| **191.9** | Malignant neoplasm of brain, unspecified | 15 |
| **232.3** | Carcinoma in situ of skin of other and unspecified parts of face | 15 |
| **198.3** | Secondary malignant neoplasm of brain and spinal cord | 14 |
| **202.10** | Mycosis fungoides, unspecified site, extranodal and solid organ sites | 14 |
| **225.0** | Benign neoplasm of brain | 14 |
| **172.3** | Malignant melanoma of skin of other and unspecified parts of face | 13 |
| **238.1** | Neoplasm of uncertain behavior of connective and other soft tissue | 13 |
| **170.9** | Malignant neoplasm of bone and articular cartilage, site unspecified | 12 |
| **195.0** | Malignant neoplasm of head, face, and neck | 12 |
| **200.30** | Marginal zone lymphoma, unspecified site, extranodal and solid organ sites | 12 |
| **205.00** | Acute myeloid leukemia, without mention of having achieved remission | 12 |
| **215.3** | Other benign neoplasm of connective and other soft tissue of lower limb, including hip | 12 |
| **218.2** | Subserous leiomyoma of uterus | 12 |
| **224.3** | Benign neoplasm of conjunctiva | 12 |
| **235.5** | Neoplasm of uncertain behavior of other and unspecified digestive organs | 12 |
| **141.9** | Malignant neoplasm of tongue, unspecified | 11 |
| **173.5** | Other and unspecified malignant neoplasm of skin of trunk, except scrotum | 11 |
| **173.81** | Basal cell carcinoma of other specified sites of skin | 11 |
| **196.0** | Secondary and unspecified malignant neoplasm of lymph nodes of head, face, and neck | 11 |
| **227.1** | Benign neoplasm of parathyroid gland | 11 |
| **239.1** | Neoplasm of unspecified nature of respiratory system | 11 |
| **146.0** | Malignant neoplasm of tonsil | 10 |
| **151.9** | Malignant neoplasm of stomach, unspecified site | 10 |
| **196.9** | Secondary and unspecified malignant neoplasm of lymph nodes, site unspecified | 10 |
| **224.0** | Benign neoplasm of eyeball, except conjunctiva, cornea, retina, and choroid | 10 |
| **225.3** | Benign neoplasm of spinal cord | 10 |
| **239.89** | Neoplasms of unspecified nature, other specified sites | 10 |

Supplementary Table 3 – ICD9 codes and descriptions of patient “cancer” encounter diagnoses that resulted in the exclusion of at least 10 patients from the primary study cohort. Counts reflect the number of unique patients in the post-intervention time period that were excluded due to the respective item. This included 5,230 total patients (note that some patients have multiple “cancer” problem list items). ICD9: International Classification of Diseases, Ninth Edition.
